# Impact of Na/Al Ratio on the Extent of Alkali-Activation Reaction: Non-linearity and Diminishing Returns

**DOI:** 10.3389/fchem.2021.806532

**Published:** 2022-01-03

**Authors:** Omar Abdelrahman, Nishant Garg

**Affiliations:** Department of Civil and Environmental Engineering, University of Illinois at Urbana-Champaign, Urbana, IL, United States

**Keywords:** alkali-activated materials, geopolymers, metakaolin, isothermal calorimetry, FTIR, XRD, NMR, TGA

## Abstract

To address the high CO_2_ footprint associated with cement production, many alternative, sustainable binders are now gaining worldwide attention–including alkali-activated materials. The alkali-activation reaction of metakaolin is a fairly complex process involving transformation of one amorphous reactant (precursor metakaolin) into another amorphous product or products (N-A-S-H gel and/or disordered zeolite type phases). In spite of this complexity, researchers in the past 2 decades have gained significant knowledge on the nature of this reaction at multiple scales. Understanding and developing a clear relationship between the alkalinity of the mix and the extent of reaction is of high interest for practical applications. However, detailed and thorough investigations on this important relationship are limited. Here, in this study, we address this gap by systematically investigating a series of alkali-activated materials samples with a wide range of Na/Al ratios (0.5–1.8) using seven different yet complementary analytical techniques (isothermal calorimetry, FTIR, XRD, TGA, NMR, and Raman imaging). Applied in tandem, these tools reveal a clear but non-linear relationship between the Na/Al ratio and the extent of alkali-activation reaction indicating diminishing returns at higher Na/Al ratios, where higher Na/Al ratios cause an increase in the degree of reaction until a certain point at which the increase in Na/Al ratio does not significantly affect the reaction kinetics, but may affect the gel polymerization. These findings could potentially aid decision making for commercial applications of AAMs where alkalinity of the mix is an important parameter for performance as well as safety.

## Introduction

The global environmental impact of the cement industry is resulting in an increasing concern and dedication towards finding more sustainable alternatives for Portland cement. One of the most promising alternatives is alkali-activated materials (AAM) (Rivera et al., 2021; van Deventer et al., 2021), also referred to as geopolymers. The terms geopolymers (Davidovits, 1989), inorganic polymers (Van Wazer, 1970), alkali-activated cement (Palomo and López de la Fuente, 2003), and geocement (Krivenko, 1994) are all used to describe these binding materials, which can provide comparable, or higher performance than the traditional cementitious binders (Wang et al., 2019; Alzeer et al., 2021) with significantly lower greenhouse emissions (Gartner, 2004; Palomo et al., 2021).

These binders are manufactured by activating an aluminosilicate precursor such as metakaolin, blast furnace slag, or fly ash using an alkaline solution, mostly sodium hydroxide or silicate solutions. The chemical composition of the activation product depends on the used raw materials. High-calcium alkali-activated materials such as slag or class C fly ash result in the formation of an aluminum-substituted C-A-S-H gel (Escalante-García et al., 2003; Fernández-Jiménez et al., 2003), with the possibility of some replacement of Ca^2+^ by Na^+^, leading to the formation of both sodium- and aluminum-substituted C-(N)-A-S-H type gel (Myers et al., 2013; Sankar et al., 2019). On the other hand, the activation of low-calcium or calcium-free materials such as metakaolin or class F fly ash results in the formation of a N-A-S-H gel, an amorphous binder that consists of a random network of tetrahedral SiO_4_ molecules linked to AlO_4_^−^ units by sharing oxygen atoms, with Na^+^ ions balancing the negative charge of the Al^3+^ in IV-fold coordination (Davidovits, 1989).

The activation process, as well as the mechanical and chemical properties of the produced binder, are dependent on several synthesis parameters such as the nature of the aluminosilicate source, the activating solution used, mix design, and curing conditions. (Rees et al., 2007; Silva et al., 2007; Granizo et al., 2014; Ghanbari et al., 2017). Another parameter that is also of equal importance for the alkali-activated binder formation is the alkalinity, which can be expressed as the molar ratio of M/Al or M/Si, where M represents the alkali ions, e.g., Na or K (De Vargas et al., 2011; Granizo et al., 2014).

Previous research has revealed to some extent the effect of the concentration of the alkaline solution on the formation process of the binders (Granizo and Blanco, 1998; Yao et al., 2009; Zhang et al., 2012; Zhang et al., 2013; Sun and Vollpracht, 2018; Hou et al., 2019; Król et al., 2019). It has been found, using various analytical techniques, that the increase in alkaline solution concentration can generally increase the reaction rate and the extent of the activation reaction. At extremely high dosage rates of alkalis, the nanoscale ordering and the polymerization of the silicate network of the C-(N)-A-S-H gel have been found to be affected (Garg et al., 2019). However, the precise effect of the activating solution’s alkalinity, which can be represented by the mix’s Na/Al ratio, on the phase assemblage and structure of the produced gel is yet to be fully understood.

In this study, a multi-technique correlative analysis was performed on alkali-activated binder samples manufactured using metakaolin as the aluminosilicate source precursor, with variable alkaline concentrations resulting in binders with a wide range of Na/Al ratios (0.5–1.8). All other parameters, such as Si/Al and water/binder ratios, were kept constant for all the mixes (1.5 and 1.0, respectively). The techniques used include isothermal calorimetry, Fourier-Transform infrared spectroscopy (FTIR), X-ray diffraction analysis (XRD), thermogravimetric analysis (TGA), ^27^Al and ^23^Na magic angle spinning solid-state nuclear magnetic resonance (MAS NMR), and Raman spectroscopy and imaging. Applying these different yet complementary techniques provides a thorough understanding of the activation reaction kinetics and the chemical and crystalline structure of the produced gel. The acquired results indicate the increasing formation rate of the N-A-S-H gel with increasing the Na/Al ratio, as well as the greater extent of the activation of the metakaolin precursor. Correlations between the different techniques were deduced by analyzing and comparing each technique’s results. Specifically, a strong correlation was found between the cumulative heat from isothermal calorimetry and the Si-O-T peak position in the FTIR data (observed in the region of 1,200–950 cm^−1^ wavenumber), suggesting that FTIR Si-O-T peak position is a good indicator of the overall alkali-activation. In addition, the alkalinity was found to affect the peak position of XRD’s zeolite phase and NMR’s Al^(4)^ coordination in a very similar manner. Also, the zeolite/anatase peak ratio was compared to NMR’s Al^(6)^/Al^(4)^ ratio, and a correlation between the two ratios was deduced, recognizing that each ratio has a different sensitivity to changes in alkalinity at different Na/Al ranges. Finally, and most importantly, all techniques pointed to the fact that there’s a non-linear relationship between Na/Al ratio and the extent of alkali-activation reaction.

## Materials and Methods

### Sample Preparation

The metakaolin used as the solid precursor to synthesize the AAM mixtures was MetaMax^®^ manufactured by BASF (New Jersey, United States). The activating solution was prepared using sodium hydroxide pellets manufactured by MACRON Fine Chemicals and sodium silicate solution (D solution by PQ^®^). To prepare the mixes, NaOH and Na_2_SiO_3_ were mixed first and left to cool down to room temperature before adding the precursor. The ingredients were mixed in a Vortex mixer for 1 minute, left to rest for 30 s, and then mixed again for another minute at a speed of 2,500 rpm. After that, the slurry was cast into a silicon mold to form cubic samples of 1 cm sides. The samples were then sealed with a parafilm, and left to cure at room temperature. At the desired age, the samples were extracted and tested. The AAM mixtures all had the same Si/Al molar ratio of 1.5 and water/binder ratio of 1.0. The Na/Al molar ratio was adjusted for the different mixtures ranging from 0.5 to 1.8. This single ratio was adjusted by manipulating the amount of NaOH added to the mix while keeping all the other components constant for all the mixes.

### Experimental Procedures

Isothermal calorimetry tests were conducted on the alkali-activated binder samples using a TAM Air isothermal calorimeter manufactured by TA Instruments, United States. The samples were all manufactured using the same precursor mass of 5 g. The samples were mixed, shaken, and then rapidly poured into plastic HDPE ampoules and placed inside the instrument. The temperature was set at 22°C, and the data were collected for the first 72 h in which both the heat flow and the cumulative heat were monitored. This duration was deemed sufficient for studying the kinetics and the extent of the reaction of the alkali-activated binders. However, the first 45 min of data were disregarded because of the disturbance in the baseline caused by the insertion of the sample holders. It was found that 45 min is the time required by the instrument to re-stabilize after this disturbance.

IR spectra were measured using a PerkinElmer FTIR Frontier spectrometer with an attached universal attenuated total reflectance (UATR) polarization accessory. The spectra were collected in the region of 4,000–400 cm^−1^ for 30 scans per sample with a spectral resolution of 4 cm^−1^. FTIR scans were performed on the constituents of the binder (metakaolin, NaOH, and PQD sodium silicate solution) and the AAM mixes having different Na/Al ratios in the range of 0.5–1.8. The samples were scanned at different time intervals, starting from 15 min after mixing up to 45 days.

X-Ray Diffraction was performed on the powdered samples at the age of 3 months to get their mineralogical composition. The instrument used was Bruker D8 Advance Plus (CuKa radiation, 40 kV, 40 mA) equipped with an EIGER2 R 500 K detector. The measuring time was 40 min per sample, with a 2θ range of 5–70° and a step size of 0.01°.

Thermogravimetric analysis tests were performed on the AAM samples at the age of 3 months using the Q50 TGA instrument by TA Instruments, United States. The samples (16–20 mg) were subjected to a temperatures range of 23–800°C at a heating rate of 10°C per minute and in a nitrogen flow of 60 ml per minute. The instrument measured the sample’s weight loss and calculated its derivative as a function of temperature. These data were used to quantify and compare the amount of the geopolymer gel formulated in each sample.

Single-pulse ^27^Al MAS solid-state NMR tests were performed on the AAM samples at the age of 4 months using a VSN750NB nuclear magnetic resonance spectrometer (Agilent, United States) with a frequency of 750 MHz (17.6 T). The powder samples were placed into 4 mm diameter rotors and installed inside the instrument’s T3 Triple Res HXY solids probe. The spinning speed was set at 13 kHz for metakaolin and 9 kHz for the AAM samples. Higher spinning speed was used for metakaolin to avoid overlapping the spinning sideband peaks with the peaks of the different Al coordination environments found in metakaolin. A total of 1,024 scans were taken for each sample with a relaxation delay time of one second. The chemical shifts were measured with respect to zero reference from an aluminum nitrate solution (Al(NO_3_)_3_). For a direct comparison, the peak intensities in the final spectra in all the figures have been normalized to the sample mass packed in the NMR rotors.

Single-pulse ^23^Na MAS solid-state NMR tests were also performed on the same AAM samples using a UI300 nuclear magnetic resonance spectrometer (Varian, USA) with a frequency of 300 MHz (7.05 T). The spinning speed was set at 10 kHz, and a total of 4,000 scans were taken for each sample with a relaxation delay time of 1 s. The chemical shifts were measured with respect to zero reference from sodium chloride solution (NaCl). The peak intensities in the final spectra in all the figures have also been normalized to the sample mass packed in the NMR rotors.

Finally, confocal Raman spectra and images were collected using a WITec Alpha 300 series SNOM confocal microscope on 7 day old AAM samples. A laser with a wavelength of 532 nm and an excitation power of 7 mW was used, with a grating of 600 g/mm coupled with a charged coupled device (CCD). The spectral resolution was 4.9 cm^−1^, and the spectra were acquired in the wavenumber range of 0–3,700 cm^−1^. The Raman spectra were collected on a 1 mm × 1 mm area with a total points number of 22,500 per image, resulting in a lateral resolution of ∼6.7 μm. A 20X objective lens was used, with a working distance of 2.2 mm and a numerical aperture of 0.5. The integration time was set to 0.1 s/point totaling around 38 min per sample for these acquisition conditions.

## Results

### Reaction Kinetics via Isothermal Calorimetry

The rate and extent of the reaction are of significant importance in describing the alkali-activation process. A calorimetric characterization technique can provide real-time information about the reaction while it is running, which has a significant advantage over other techniques that acquire data in a discontinuous manner (Provis et al., 2005; Khale and Chaudhary, 2007). Isothermal calorimetry provides the heat flow rate during the reaction process, which serves as an indication for the reaction rate, and the cumulative emitted heat, which can represent the amount of metakaolin that has reacted in each of the different mixes. Recently, isothermal calorimetry has been successfully employed to study the reaction kinetics of alkali-activated slags, where it has been found highly complementary to other advanced X-ray (Garg and White, 2017) and Neutron scattering analysis (Gong et al., 2019).

**FIGURE 1:**
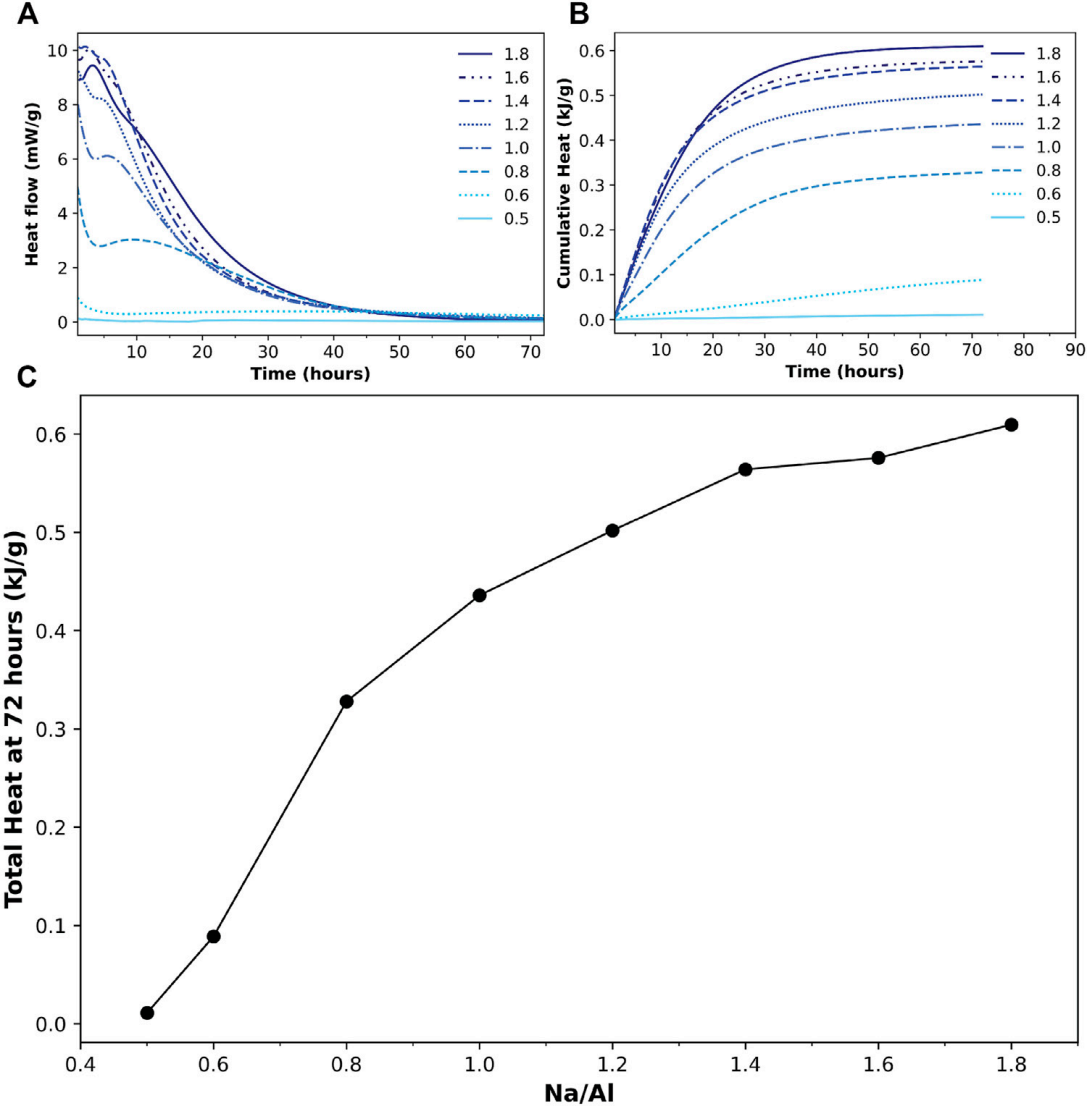
**(A)** Heat flow and **(B)** cumulative heat results from isothermal calorimetry tests for 72 h on alkali-activated metakaolin samples with various Na/Al ratios, indicated in figure (0.5–1.8). **(C)** The total heat generated after 72 h of activation for different Na/Al ratios. The line is a guide to the eye.

Here, isothermal calorimetry was performed on the AAM samples for 72 h, which can be considered sufficient to monitor the fundamental region of the alkali-activation process. Figure 1 shows the acquired results for all the samples. The recorded heat flow is shown in Figure 1A, in which two exothermal peaks can be observed. The initial exothermal peak, which typically appears as soon as the alkaline solution is added to the precursor, is not fully observable here as the samples were already mixed before placing them in the calorimeter, and also because of the disregard of the first 45 min of data until the baseline was stabilized. However, the decline of this peak can be clearly observed in the first few minutes of the recorded data.

A second exothermic peak is observed sometime later than the first one, ranging from just a few minutes for the samples with high Na/Al ratios to more than 10 hours for those with relatively lower ratios, which is similar to what is observed in previous literature (Yao et al., 2009; Zhang et al., 2013). As for the two samples with the lowest Na/Al ratios (0.6 and 0.5), the two peaks are not fully observed. Only the decline of the first peak can be seen in the 0.6 sample, without a trace of a second peak. The 0.5 sample, however, does not show any of the peaks at all, indicating very little activation reaction occurring in the mix.

The cumulative heat generated by the reaction in the samples is plotted in Figure 1B. The sample with the lowest Na/Al ratio of 0.5 resulted in very little overall cumulative heat. As the Na/Al ratio increased up to 1.8, the cumulative generated heat increased accordingly. A correlation between the Na/Al ratio and the total cumulative heat after 72 h of reaction is plotted in Figure 1C. This plot clearly shows the increasing cumulative heat with the increase of the mixes’ alkalinity, which indicates a greater extent of alkali-activation reaction as the Na/Al ratio is increased.

### Reaction Kinetics via FTIR

Infrared spectroscopic techniques have been widely used to study different aluminosilicate structures. FTIR has proven to be very successful in identifying the different zeolite structures based on the analysis of molecules vibration (Mozgawa, 2001), which makes this method useful in analyzing materials with amorphous phases (Król et al., 2012).

**FIGURE 2:**
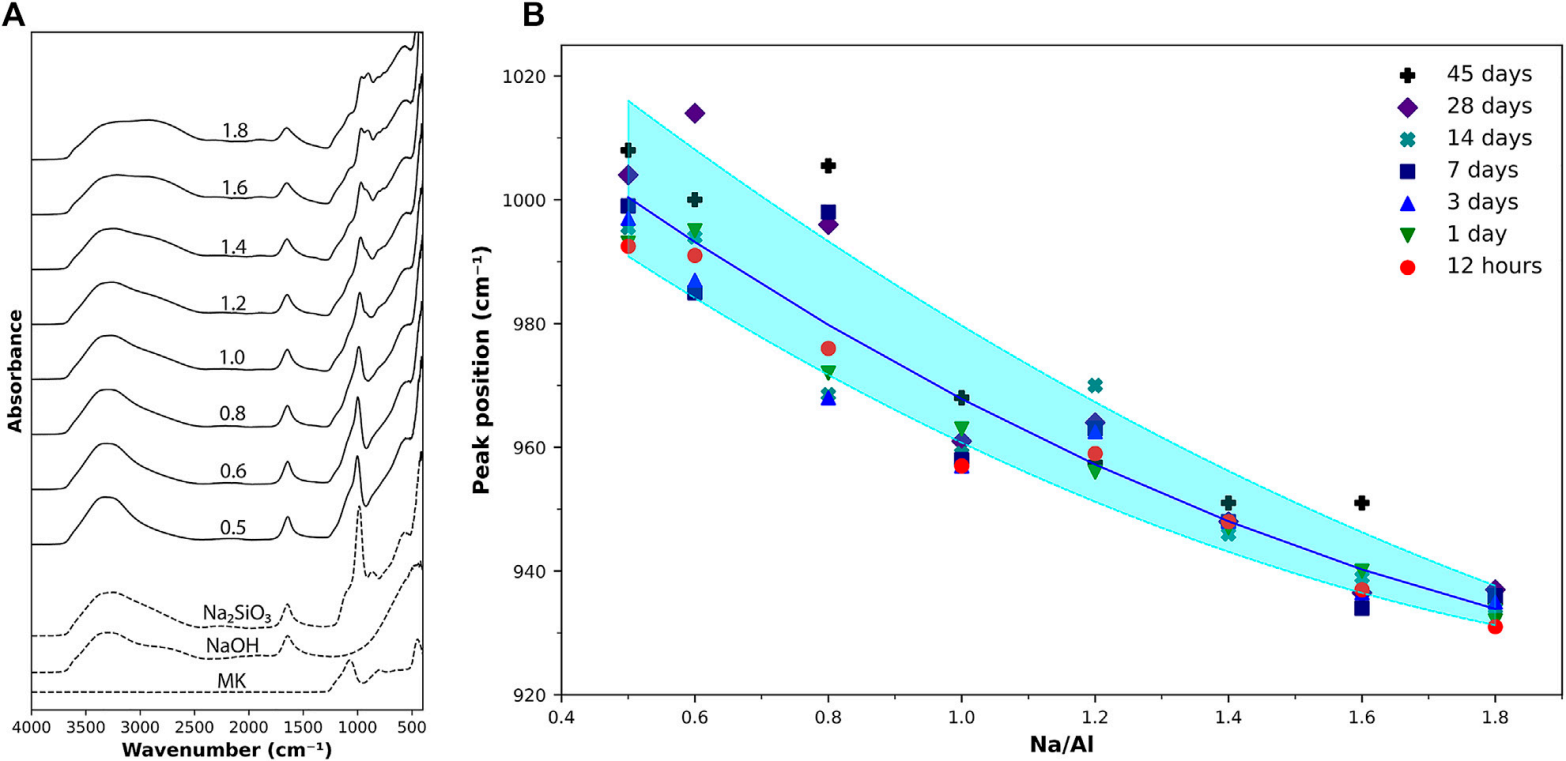
**(A)** FTIR scan results on metakaolin, activating solutions, and AAM samples with various Na/Al ratios (as listed) scanned 15 min after activation. **(B)** Si-O-T peak position for various Na/Al ratios at different ages. The shaded region provides a guide to the eye of how the Na/Al ratio affects the peak position.

The results of FTIR scans on various AAM samples are presented in Figure 2. The FTIR spectra of the main constituents of the mixes (metakaolin, NaOH, and PQD sodium silicate solution) along with the AAM samples of Na/Al ratios of 0.5–1.8, scanned 15 min after mixing, are shown in Figure 2A. The bands in the range of 1,100–400 cm^−1^ represent the aluminosilicate structure of the material. A specific peak of interest is the peak in the region of 1,200–950 cm^−1^ observed in metakaolin and in all the AAM samples. This peak represents the primary Si-O-T asymmetric stretching vibration (Stubičan and Roy, 1961; Król et al., 2019; Biel et al., 2020).

In the unreacted metakaolin powder, this peak is located at a wavenumber of 1,071 cm^−1^. After activation, it is found that the band gradually shifts towards more negative wavenumber values as the Na/Al ratio increases, ranging from 1,014 cm^−1^ at a Na/Al ratio of 0.6–931 cm^−1^ at a Na/Al ratio of 1.8. Figure 2B shows the position of this peak as a function of the Na/Al ratio for all the tested samples at different ages. This negative shift of the band position in the high Na/Al ratio mixes is the result of the lower presence of component bands at the higher wavenumbers, which are associated with the amount of unreacted metakaolin present in the sample (Król et al., 2016). Thus, the shift in the Si-O-T peak position can serve as an indication of the extent of the alkali-activation reaction occurring in each sample, showing again, a clear increase in the reaction as the alkalinity of the mix increases.

### Phase Assemblage via XRD

X-Ray Diffractometry (XRD) is a crucial technique for detecting alkali-activated binders’ mineralogical phase assemblage. The zeolitic binder phase formed by the alkali-activation of metakaolin has been often described as “X-Ray amorphous” (Barbosa et al., 2000; Provis et al., 2005). Studies have shown that the primary growth units during the zeolite formation are particles around 2–5 nm in size (Nikolakis et al., 2000; Kragten et al., 2003). This length scale is below the detection limit of XRD. That is why no observable peaks are usually detected on the XRD spectrum of zeolitic geopolymers. However, a broad hump, centered at around 27–29° 2θ, is a typical feature observed in the XRD diffraction pattern of alkali-activated materials. (Alonso and Palomo, 2001; Rowles and O’Connor, 2003; Yunsheng et al., 2010; Li et al., 2019; Alventosa and White, 2021). This peak has been considered the distinguishing feature of geopolymers as it was present in any geopolymer binder regardless of the type of precursor used, activating solution, and curing conditions (Provis et al., 2005).

**FIGURE 3:**
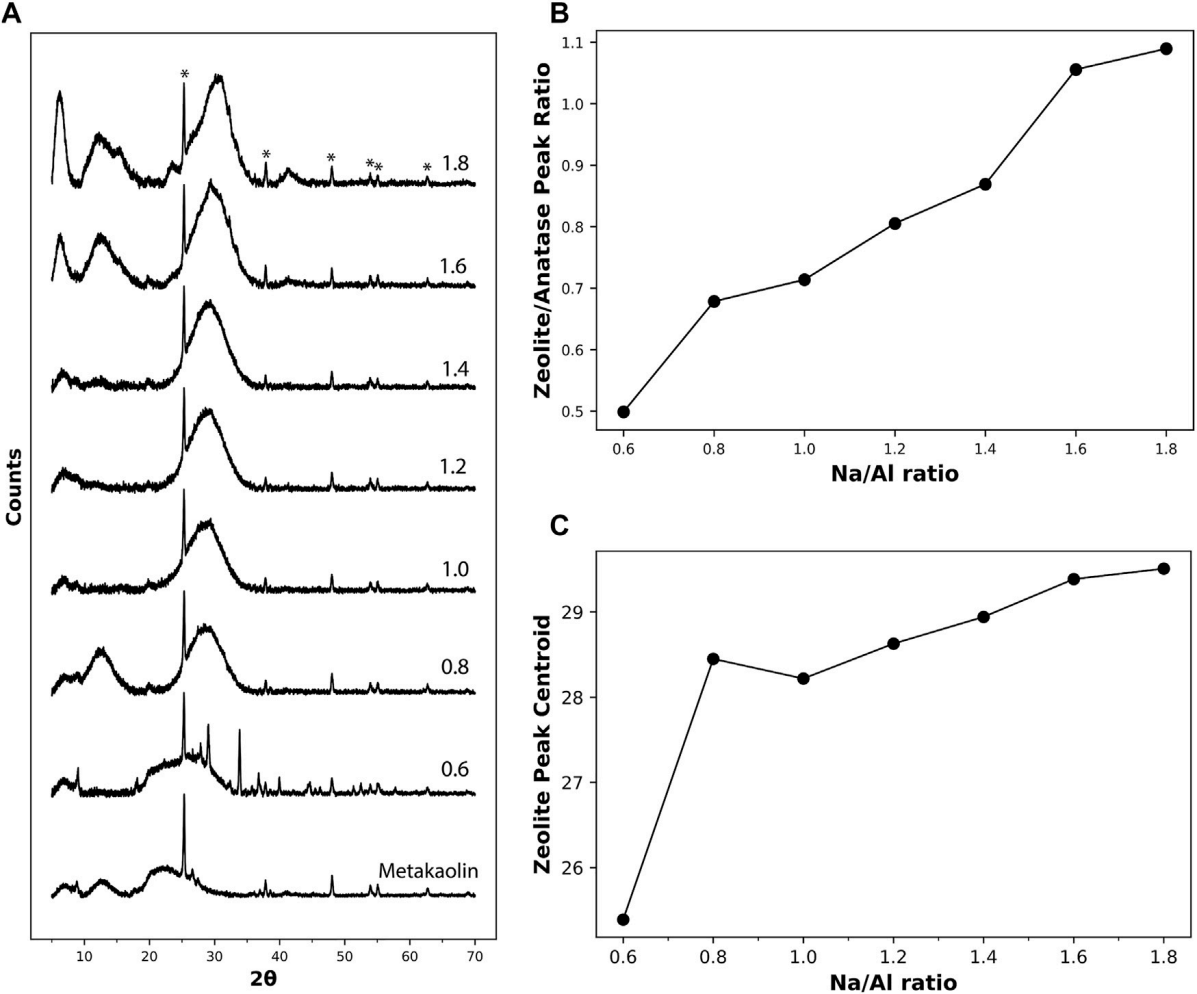
**(A)** XRD diffractograms of metakaolin precursor and the different 3 month old AAM samples with Na/Al ratios ranging from 0.6 to 1.8. **(B)** The ratio between the height of the zeolite XRD hump at 2θ ≈ 29^o^ and the anatase’s prominent peak at 2θ ≈ 25° for the different samples. **(C)** The location of the centroid of the peak representing zeolites for the different Na/Al ratios. The peaks marked with (*) represent anatase (TiO_2_, PDF number 01-076-0325).

The diffraction patterns of metakaolin and the 3 month old AAM powders having different Na/Al ratios are plotted in Figure 3A. The metakaolin spectrum shows a highly amorphous structure, except for a highly distinctive peak at an angle 2θ of around 25°, along with a series of smaller distributed peaks. These peaks match the exact spectrum of anatase (TiO_2_, PDF number 01-076-0325) (Howard et al., 1991), which is present as an impurity in the metakaolin precursor. Despite the modest anatase content inside the metakaolin, it highly dominates the XRD spectrum because of its highly crystalline structure (Thamaphat et al., 2008). In addition to these peaks, the diffractogram of unreacted metakaolin shows a broad amorphous hump at around 22° 2θ. This hump is also observed in the AAM samples having very low Na/Al ratios, indicating the presence of high amounts of unreacted metakaolin. This observation is in good agreement with previous research work in AAM mixes that had low quantities of activating solution used, causing only partial activation of metakaolin (Rowles and O’Connor, 2003).

For the XRD spectrum of the alkali-activated metakaolin, the anatase peaks are also observed after activation since they are not involved in the reaction process. The hump representing zeolites and/or a N-A-S-H type gel can be observed in the 2θ range of 27–29°. It is observed that the height of the hump increases with the increase in the Na/Al ratio, accompanied by a decrease in its width. The increase in the height of the zeolite peak, normalized to the anatase peak, with increasing alkalinity is plotted in Figure 3B. This peak sharpening effect, which was also observed in previous XRD analysis on AAMs (Yang et al., 2000; Zhan et al., 2002), is due to an increased level of crystallization within the product gel, causing the formation of larger crystals that are easier to detect by diffraction.

In addition to the change in the hump’s sharpness, it is also observed that the hump is shifting gradually towards higher 2θ angles as the Na/Al ratio increases, as shown in Figure 3C. This behavior was also observed in previous work (Williams et al., 2011; Hou et al., 2019). In low Na/Al ratios, the centroid of the zeolite peak is at a relatively low 2θ angle due to the limited activation reaction that occurred in the mix and the presence of considerable amounts of unreacted metakaolin. This unreacted metakaolin forms a high peak component at around 22° 2θ, pulling the centroid of the hump towards lower 2θ values. For higher Na/Al ratios, a higher amount of metakaolin is activated, causing the metakaolin component to go down and the centroid of the hump to shift towards higher 2θ values. Thus, based on this observed behavior, the centroid location of the zeolite and/or N-A-S-H gel hump could serve as an indication of the extent of the alkali-activation reaction.

### Phase Assemblage via TGA

Thermogravimetric Analysis (TGA) has been used to quantify the amount of hydration products of different binders, which can be achieved by tracking the weight loss of the sample when subjected to an elevating temperature. The weight-loss behavior can be attributed to the amount of bound water inside the binder structure, where the weight loss percentage can represent the weight of water present, and the derivative of weight loss (DTG) can be used to identify the source of the water that is evaporating inside the sample (Nath et al., 2016). Previous research has identified the weight loss of water bound inside the N-A-S-H gel to be in the region between 85–135°C (Duxson et al., 2007b; Winnefeld et al., 2010; Rocha et al., 2018; Chaipanich et al., 2019).

**FIGURE 4:**
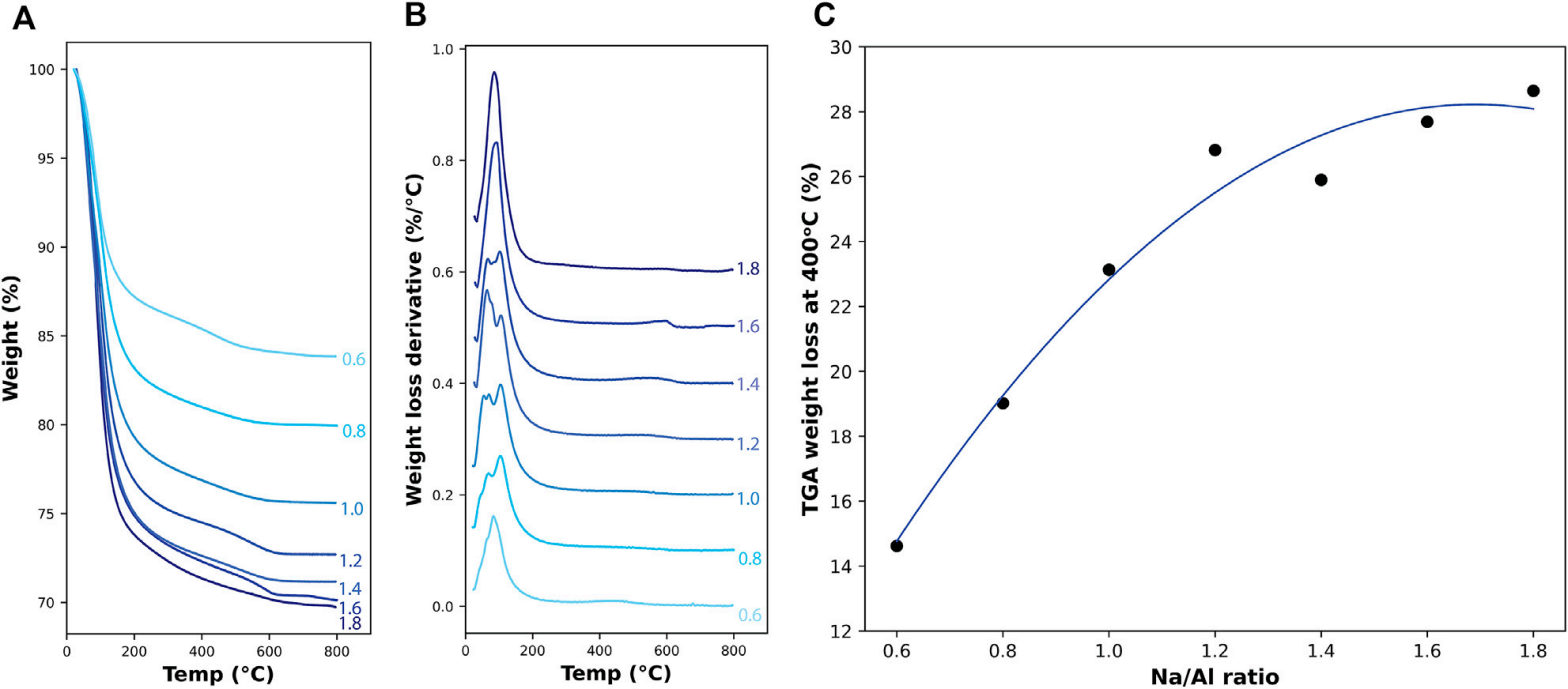
TGA results on 3 month old AAM samples. **(A)** shows the percentage of weight loss and **(B)** shows the derivative weight loss with increasing temperature up to 800°C. **(C)** shows the total percentage of weight lost in the samples at a temperature of 400°C for different Na/Al ratios, with a second-degree polynomial best fit curve.

TGA and DTG results of the 3 month old AAM samples are plotted in Figure 4. The weight loss percentage is shown in Figure 4A along with each sample’s Na/Al ratio, and Figure 4B shows the derivative weight loss for the same samples. A direct relationship between the Na/Al and the weight loss at a temperature of 400°C is shown in Figure 4C. Temperatures above 400°C were not considered for this plot to remove any weight loss that could have occurred due to the presence of carbonation products that could have formed inside the samples. Weight loss of carbonation products is usually observed at temperatures of around 600°C (Abdalqader et al., 2015). The graph shows that the increase in Na/Al ratio from 0.6 to 1.8 is accompanied by an increase in weight loss from 14.6 to 28.6%. The higher weight loss can be attributed to the increase in the bound water inside the binder structure, which supports the finding that the amount of N-A-S-H gel increases with increasing Na/Al ratio.

### ^27^Al Local Environments via MAS NMR

The molecular arrangements of the alkali-activated binders can be studied with solid-state MAS NMR, which has been proven to be of great value for the study of alkali-activated metakaolin in particular (Provis and Deventer, 2009). It was one of the first advanced analytical techniques to be applied for the study of alkali-activated metakaolin (Davidovits, 1988) and has become a widely used technique ever since (Barbosa et al., 2000; Singh et al., 2005; Rowles et al., 2007; Ruiz-Santaquiteria et al., 2012, 2013).

^27^Al MAS NMR results can be used to indicate the extent of reaction of metakaolin into geopolymer gel (Provis and Deventer, 2009). The coordination of Al varies with the change of the number of oxygen atoms immediately bonded to each Al position. This results in Al coordination environments of 4, 5, and 6. Since the Loewenstein avoidance principle (Loewenstein, 1954) states that Al-O-Al bonds cannot exist, it is safe to assume that the coordination number refers to the number of SiO_4_ tetrahedra that can connect to an AlO_n_ moiety, as only silicon can be the next-nearest neighbor (Rowles et al., 2007). The ^27^Al chemical shifts defining Al^(4)^, Al^(5)^, and Al^(6)^ are well separated and allow for the coordination number of AlO_n_ units to be clearly determined (Rowles et al., 2007; Vinet and Zhedanov, 2011).

**FIGURE 5:**
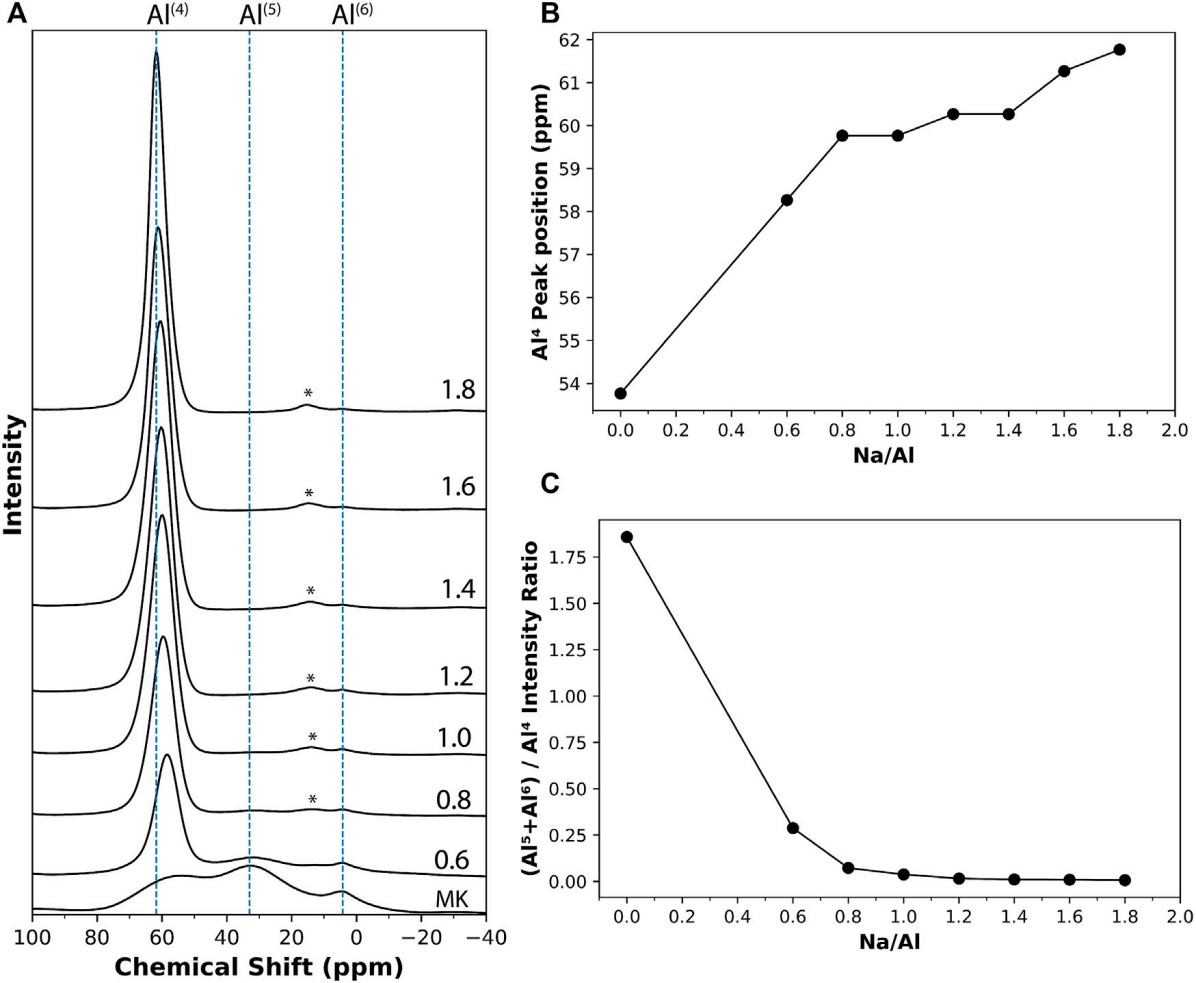
**(A)** ^27^Al solid-state MAS NMR results on metakaolin and different 4 month old AAM samples with varying Na/Al ratios (0.6–1.8), identified on each plot. The chemical shift values are referenced to Al(NO_3_)_3_ solution. The results were normalized according to the samples’ weights. The peaks marked with (*) are spinning sidebands. **(B)** The change in the position of Al^(4)^ peak with respect to the Na/Al ratio. **(C)** The Al^(5)^+Al^(6)^ to Al^(4)^ peak ratio for the different mixes.

The results of ^27^Al MAS NMR scans on metakaolin precursor exhibited three broad resonances at 54, 33, and 4.2 ppm, as seen in Figure 5A, which are assigned to Al^(4)^, Al^(5)^, and Al^(6)^ coordination environments, respectively (Rocha, 1999; Duxson et al., 2007a; Fernandez et al., 2011). The presence of multiple coordination environments in metakaolin and its amorphous nature enhances its reactivity and helps initiate the activation reaction (Granizo et al., 2000). Specifically, Al^(5)^ sites have been recently reported to contribute significantly to the reactivity and dissolution of metakaolin (Garg and Skibsted, 2019). After the reaction, however, the ^27^Al MAS NMR results for the 4 month old alkali-activated metakaolin samples are dominated by a single resonance in the 58–62 ppm range (Figure 5A), suggesting an almost complete transformation from the Al distribution observed in metakaolin to only Al^(4)^ coordination. A negative shift was observed in the Al^(4)^ resonance in the series of ^27^Al MAS NMR spectra of the alkali-activated metakaolin with varying Na/Al ratios. The Al^(4)^ resonance peak shifted from 58.3 ppm for Na/Al ratio of 0.6, to 61.8 ppm for Na/Al ratio of 1.8. This relationship between the Na/Al ratio and the position of the Al^(4)^ resonance peak is presented in Figure 5B. This negative shift is similar to what is found in recent work (Balzer et al., 2020), and it can be explained by the re-arrangement of the tetrahedral units after the hydrolysis of the T–O–T bonds and the formation of more Q^3^AlOH, which has a higher ppm value than the Q^4^ Al (Zeng et al., 2000).

Figure 5A also shows low-intensity resonance peaks representing Al^(5)^ and Al^(6)^ coordination that can still be observed in the mixes with lower Na/Al ratios. Their intensity decreases as the Na/Al ratio increases until they disappear at high-Na mixes. These peaks serve as an indication of the amount of unreacted metakaolin in the mix (Duxson et al., 2005; Rowles et al., 2007). Thus, a ratio of the peak intensity of (Al^(5)^+Al^(6)^) to that of Al^(4)^ can be used to estimate the relative amount of unreacted metakaolin in each mix. This ratio is plotted in Figure 5C with respect to each mix’s Na/Al ratio. A clear trend that can be observed which shows the decrease in unreacted metakaolin with the increase of Na/Al ratio.

### ^23^Na MAS NMR

Since Na^+^ ions are incorporated into the alkali-activated systems and the formulated NASH gel, the characterization of the ^23^Na environment can provide additional valuable information about the extent of alkali-activation reactions of the different systems. Previous literature has identified that the bond of Na in AAM gel is in the form of Na(H_2_O)_n_^+^, where n = 2–8 (Škvára et al., 2009). However, the Na-O bonds are more ionic and much weaker than Si-O or A1-O bonds, and thus, the Na coordinations are often believed to be ill-defined (Xue and Stebbins, 1993). This results in a broad peak, as observed by previous researchers testing ^23^Na NMR resonance on AAMs (Duxson et al., 2005; Rowles et al., 2007; Walkley et al., 2016).

**FIGURE 6:**
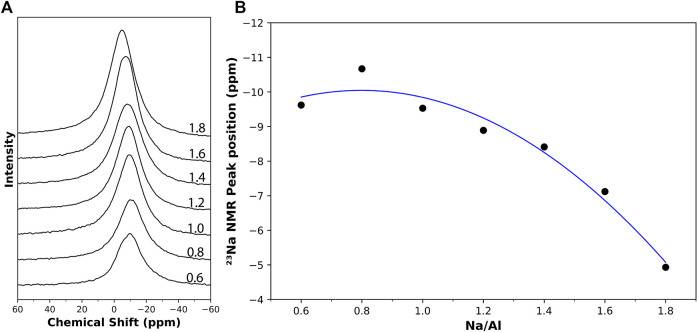
**(A)** ^23^Na solid-state MAS NMR results on 4 month old AAM samples with varying Na/Al ratios (0.6–1.8), identified on each plot. **(B)** The change in the ^23^Na NMR peak position with respect to the Na/Al ratio.

The results obtained from the ^23^Na NMR tests on the AAM samples showed a single broad peak, as seen in Figure 6A. The position of this peak is seen to be shifting with the change in the Na/Al ratio, ranging between -9.62 ppm and -4.93 ppm for Na/Al ratios of 0.6 and 1.8, respectively. Similar behavior was observed in previous research in which the peak position was found to shift with changes in the NaOH concentration (Rowles et al., 2007). The relationship between the peak position and the Na/Al ratio is plotted in Figure 6B. It is believed that this shift can be attributed to the degree of polymerization of the structure, as the peak position is affected by the mean N-O bond length and by the number of non-bridging oxygens per tetrahedrally coordinated cation (NBO/T) (Xue and Stebbins, 1993). So, the increase in the ^23^Na peak position observed with increasing the Na/Al ratio potentially suggests that the mean Na-O bond length decreases and the NBO/T increases. Thus, although the extent of reaction increases with increasing the Na/Al ratio, the N-A-S-H gel is likely becoming less polymerized, which could lead to a microstructure that is more susceptible to leaching. Recent research has also observed similar behaviors for C-N-A-S-H gel where increasing alkalinity of the activator results in lower gel polymerization (Garg et al., 2019). That is why it is crucial to determine an optimum Na/Al ratio needed for AAM mixes to obtain both a high degree of reaction as well as an adequately polymerized structure.

### Spatial Phase Mapping via Raman Spectroscopy & Imaging

Unlike FTIR or NMR, Raman spectroscopy, so far, has been rarely employed in investigating or describing the structure of the formed gels (Caggiani et al., 2021). Analyzing AAMs using Raman spectroscopy is a quite challenging task because this material exhibits high fluorescence levels when short laser wavelengths are used. Using longer wavelengths can ameliorate the problem; however, it can also result in photoluminescence, probably due to the trace rare earth elements in metakaolin (Torréns-Martín et al., 2013b; Garg et al., 2013; Szechyńska-Hebda et al., 2019).

**FIGURE 7:**
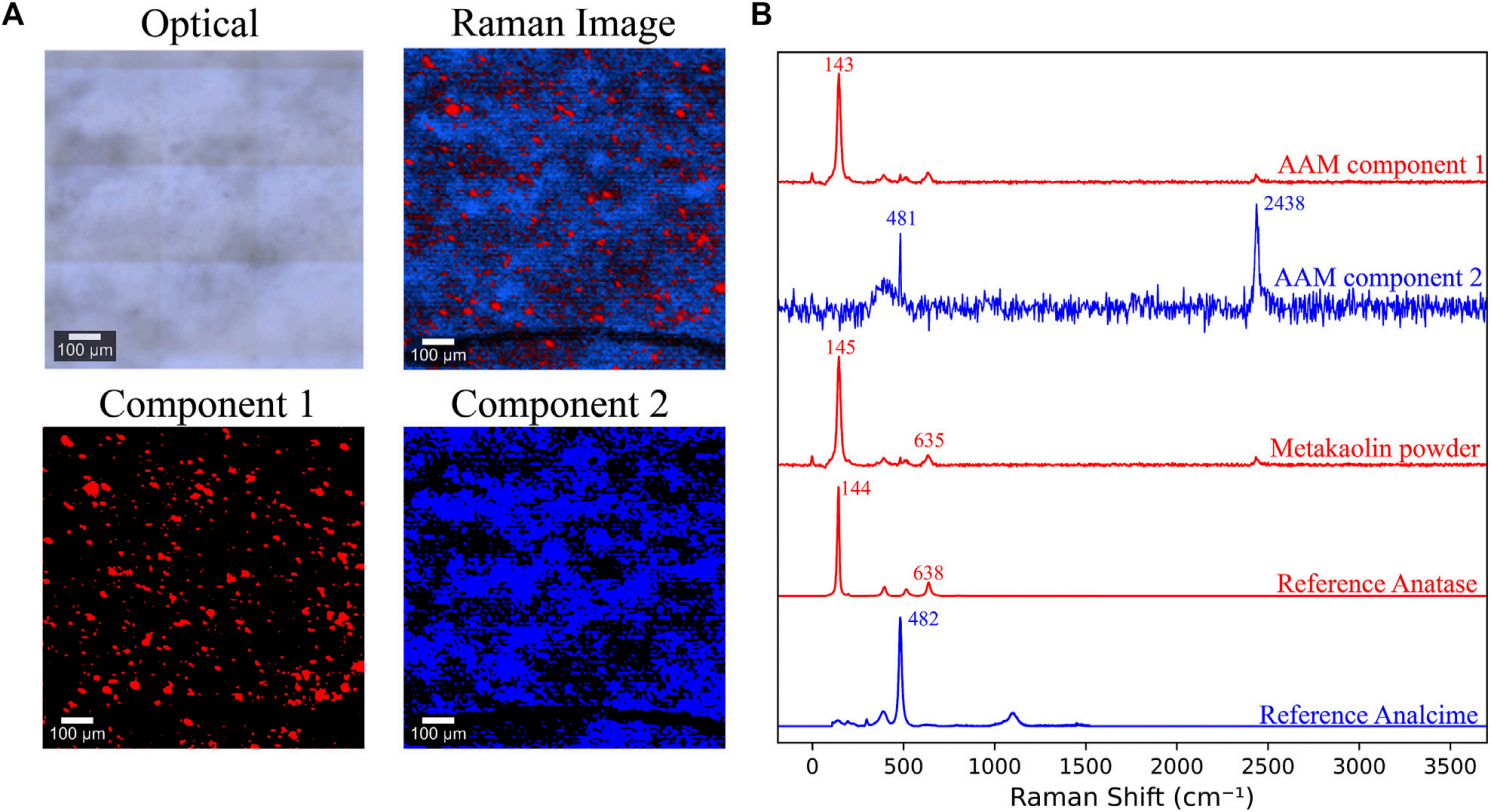
**(A)** Optical image, combined Raman image, and components maps of a 7 day old AAM sample **(B)** Raman spectra of AAM’s components, raw metakaolin powder, reference Anatase (TiO_2_) (Zhang et al., 2000), and reference Analcime (Na(AlSi_2_O_6_)·H2O) (Presser et al., 2008; Tsai et al., 2021).

Raman spectroscopy and imaging were used in this study with the aim of recognizing the spectrum representing metakaolin and then mapping and quantifying the presence of this spectrum in the AAM scans. Recently, Raman imaging has been instrumental in understanding various complex, heterogeneous systems such as granites (Polavaram and Garg, 2021b), pure cement phases (Potgieter-Vermaak et al., 2006), phase quantification in anhydrous cements (Polavaram and Garg, 2021a), hydrating cements (Loh et al., 2021), calcium aluminate cements (Black et al., 2006; Torréns-Martín et al., 2013a), and sulfate-attacked cementitious materials (Yue et al., 2013). The purpose of applying Raman spectroscopy and imaging here was to estimate the amounts of metakaolin that remained unreacted and thus, estimate the extent of the alkali-activation reaction in mixes with varying alkalinity. A sample of the Raman images and component spectra of a 7 day old AAM sample is shown in Figure 7. Two components were identified in the acquired spectrum. The first component had peaks at 143, 393, and 638 cm^−1^, while the second one had a peak at around 481 cm^−1^. The peak present at 2,438 cm^−1^ was recognized as an artifact in the imaging procedure, potentially attributed to an external light source in the room. The spectrum acquired from scanning metakaolin is shown in Figure 7B, also having the peaks at 143, 393, and 638 cm^−1^.

When the spectrum obtained from metakaolin was mapped in the scans of activated samples having different Na/Al ratios and compared, no recognizable or systematic variance in its amount was observed between the mixes. This does not coincide with the expected decrease in unreacted metakaolin content in the higher alkaline mixes, which means that this spectrum probably does not directly represent metakaolin. That being said, this observation does not rule out future detailed investigations of these systems with Raman imaging where more information can be extracted by optimizing the experimental parameters for these specific samples.

Some previous Raman spectroscopy research done on AAM have attributed the bands at 143, 400, 514-520, and 635–640 cm^−1^ to intratetrahedral vibrations of polymerized silicate tetrahedra, Si-O-T bending, and T-O symmetric stretching modes, respectively (Zhang et al., 2017; Brylewska et al., 2018). However, we believe that these four signals are primarily caused by the anatase present in the raw materials, which has the spectrum shown in Figure 7B (Zhang et al., 2000). This observation was also recognized by other previous researchers (Murad, 1997; Caggiani et al., 2021). In spite of being a minor impurity in metakaolin, the high detection of anatase is due to its exceptionally high cross-section for Raman scattering. This is caused by its molecular transition polarizability and its complex conjugate (Clark et al., 2007; Nakamoto, 2008). Anatase’s dominance over the Raman spectra of both metakaolin and the activated AAM constructs a significant draw-back for analyzing these materials using this technique, as it obstructs the ability to observe the other contributions and can lead to misinterpretations (Caggiani et al., 2021). Future investigations will need to focus on highly pure samples as even trace amounts of Anatase can dominate the analytical results.

The second component observed in the AAM spectrum had an observable peak at around 481 cm^−1^. This peak can be attributed to analcime, one of the species for singly connected four-ring-chain zeolites, having a composition of NaSi_2_AlO_6_·H_2_O (Tsai et al., 2021). The Raman spectrum of analcime is also shown in Figure 7B (Presser et al., 2008; Tsai et al., 2021). Analcime can be considered a representative for the formed N-A-S-H gel; however, it cannot be mapped as a reference for the amount of formed gel as it is not the only phase formed by the alkali-activation process. Due to the highly amorphous structure of the gel, it was very challenging to detect any other gel phases, leading to difficulty quantifying or comparing the amount of formed gel in the different mixes. Given the challenges noted, efforts need to continue to resolve AAM phases with Raman imaging in future studies. The detection of an analcime type phase via Raman is potentially novel and more efforts could be dedicated in this direction.

## Discussion and Broader Impacts

By assembling and cross-comparing the data acquired from all the different yet complementary techniques used in this research study, various interesting and novel correlations were found. For instance, the practicality of using FTIR to measure the rate of hydration was evaluated compared to that of isothermal calorimetry. And results acquired from structure-indicating techniques like XRD and NMR were compared to find how well they correlate in identifying the change in structure with changes in Na/Al ratio.

**FIGURE 8:**
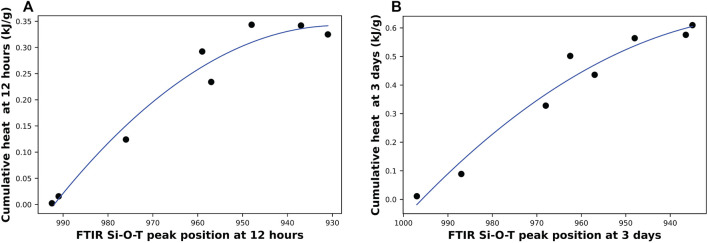
A correlation between the total heat generated from isothermal calorimetry and the position of the Si-O-T peak from FTIR scans after **(A)** 12 h of hydration and **(B)** 3 days of hydration. Each data point represents a different Na/Al ratio (0.6–1.8).

Firstly, a correlation between results acquired from isothermal calorimetry and FTIR scanning at two different times (12 h and 3 days) is plotted in Figure 8. Interestingly, a direct relationship was found between the cumulative heat of hydration and the position of the peak representing the Si-O-T bonds, where each data point represents a sample with a different Na/Al ratio. As the Na/Al ratio of the mix increases, the cumulative heat increases, and the location of the peak shifts toward lower wavenumber values. This correlation was tested at two different time points: the first one after 12 h of activation, shown in Figure 8A, and the other one after 3 days of activation, shown in Figure 8B. Both figures show that the extent of the alkali-activation reaction affects the cumulative heat and the peak position at a similar rate, and the relationship between the two techniques’ data is the same even at different time points. This correlation suggests that both techniques can almost equally monitor the amount of reaction at any point in time. This explains why most of the previous research done on AAM has been able to successfully use isothermal calorimetry and FTIR to measure the kinetics of the alkali-activation reaction (Granizo et al., 2002; Provis et al., 2005; Król et al., 2019).

**FIGURE 9:**
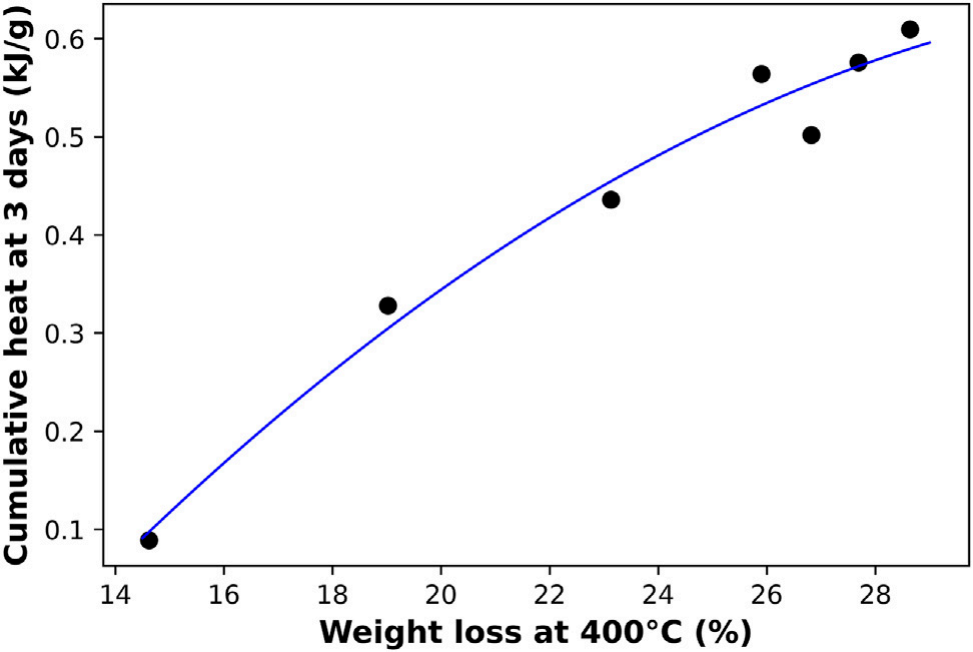
A correlation between the weight loss percentage acquired from TGA tests at 400°C on 3 month old AAM samples and the cumulative heat obtained by isothermal calorimetry after 3 days of reaction. Each data point represents a different Na/Al ratio (0.6–1.8).

TGA is another technique that was used to track the degree of reaction for each mix. Previous researchers have used it along with isothermal calorimetry to measure the extent of hydration of cementitious materials (Pane and Hansen, 2005; Deboucha et al., 2017). And since this degree of reaction can be represented by both techniques, a possible correlation between TGA’s and the isothermal calorimetry’s data was investigated and plotted in Figure 9. Again, we observe a close relationship between TGA’s weight loss percentage at a temperature of 400°C for 3 month old samples and the cumulative heat obtained by isothermal calorimetry after 3 days of alkali-activation. The correlation between both measurements indicated a strong equivalence between both techniques as they have almost identical sensitivity to the Na/Al ratio variation. Although isothermal calorimetry is considered the most reliable method to measure the degree of the activation reaction, it requires monitoring the samples in the first 45 h after hydration. Based on this correlation, it is possible to also use TGA to provide a close estimation for the extent of hydration for the already activated and hardened mixes.

**FIGURE 10:**
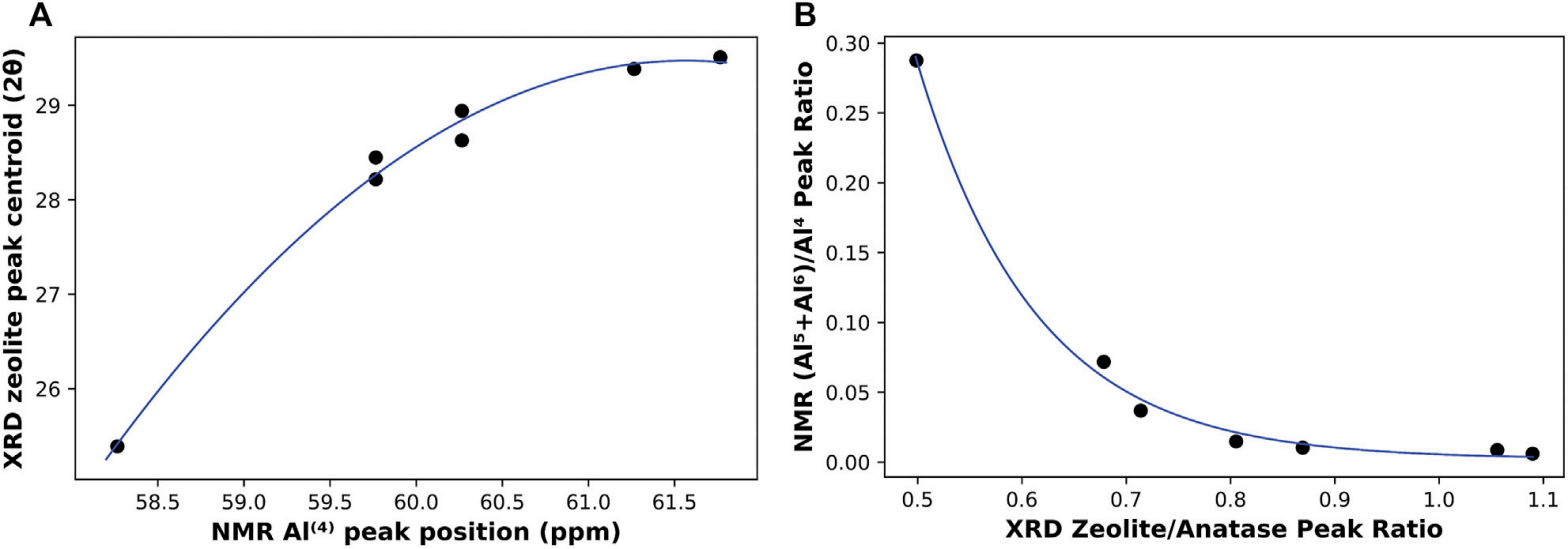
A correlation between NMR (4 month old samples) and XRD (3 month old samples) results. **(A)** shows the relationship between Al^(4)^ peak position (ppm) referenced to Al(NO_3_)_3_ solution and XRD centroid of zeolite hump (2θ), and **(B)** shows the ratio of NMR’s (Al^(5)^+Al^(6)^)/Al^(4)^ compared to the ratio of zeolite/anatase peaks from XRD.

On the other hand, changes in the structure of the activation products were monitored by both NMR (on 4 month old samples) and XRD (3 month old samples). In ^27^Al NMR, the position of the resonance peak representing the Al^(4)^ coordination was observed to be shifting towards higher frequency with the increase in Na/Al ratio, potentially indicating less shielded Al sites in the N-A-S-H gel structure (Zeng et al., 2000). As for XRD, the centroid of the zeolite peak was also found to be shifting towards higher 2θ values and away from the original hump observed in the metakaolin precursor. The rates of shifts in these peaks represent the rate of change in binder structure with respect to the Na/Al ratio. A correlation between the peaks positions of NMR’s Al^(4)^ coordination and XRD’s zeolite is plotted in Figure 10A. This plot shows the similarity in the rate of structure change identified by both techniques, particularly the considerable leap that occurred in the binder structure between Na/Al ratios of 0.6 and 0.8. This leap was almost equally recognized by both techniques.

Another way to indicate the extent of a reaction is by comparing a parameter that resembles the reaction product with another constant or less affected one. This method was applied both in XRD and NMR. In XRD, the increase in alkali-activation reaction results in an increase of zeolite and/or N-A-S-H gel formation, causing its hump to increase in size (Provis et al., 2005), while the other distinctive peak, representing anatase, remains constant, as it is not involved in any chemical reactions. Thus, a ratio of zeolite peak height to that of anatase can indicate the extent of reaction that took place in each mix. As for NMR, the reaction leads to the transformation of Al^(5)^ and Al^(6)^ coordination environments into Al^(4)^, so a higher degree of reaction should result in a lower ratio of (Al^(5)^+Al^(6)^)/Al^(4)^. That is why this ratio can be considered a representative of the unreacted metakaolin, and so, another indication of the extent of the alkali-activation reaction. These two ratios from both techniques are plotted together in Figure 10B. It is observed that although both ratios have a clear trend with Na/Al ratio, the rate of change of both ratios is slightly different. Both ratios are still pretty successful in indicating the extent of alkali-activation with the change in the Na/Al ratio. Overall, these widely different techniques are found to heavily complement each other in unambiguously finding evidence for a higher extent of alkali-activation reaction with increasing Na/Al ratio. However, the relationship between extent of reaction and Na/Al ratio is not perfectly linear and instead shows a parabolic behavior with diminishing returns. That is, at very high Na/Al ratios the reactants and products reach a saturation stage where the increase extent of reaction is not linearly proportional to the increase in Na/Al ratio, suggesting a practical upper limit to the alkalinity of the activator to be used for commercial applications.

The ^23^Na NMR results showed a shifting peak position towards more positive chemical shift values with the increase in Na/Al ratios, indicating more NBO/T in the structure and a potentially less polymerized gel structure. It is also observed in Figure 6B that these shifts are not noticeable for low Na/Al ratios, and they get more significant at the upper range of Na/Al ratios. These results also demonstrate the diminishing returns for high Na/Al ratios in AAMs.

## Conclusion

A multi-technique investigation was performed in this study on alkali-activated metakaolin mixes to investigate the effect of Na/Al ratio on the alkali-activation reaction kinetics and the final phase assemblage using a wide range of Na/Al ratios of 0.5–1.8. The reaction kinetics were studied using isothermal calorimetry, FTIR, and TGA. The isothermal calorimetry tests indicated higher heat flow and cumulative heat for higher alkalinity mixes, ranging from 0.01 kJ/g to 0.69 kJ/g for Na/Al ratios of 0.5 and 1.8, respectively. This increase in cumulative heat indicates a higher rate and extent of activation reaction for higher Na/Al ratio mixes. This result was also supported by the negative shift of the Si-O-T band in the AAM samples’ FTIR spectra. The Si-O-T band position shifted from 997 cm^−1^ for Na/Al of 0.5–935 cm^−1^ for Na/Al ratio of 1.8, indicating lower amounts of unreacted metakaolin in the high alkalinity mixes. Finally, TGA results on 3 month old AAM samples showed an increasing weight loss percentage, from 14.6 to 28.6%, by increasing the Na/Al ratio from 0.6 to 1.8, which confirmed the increase in the amount of the N-A-S-H gel inside the samples.

The phase assemblage in these AAM samples was also analyzed using XRD, NMR, and Raman spectroscopy and imaging. The increase in the Na/Al ratio resulted in an increasing peak-sharpening in addition to a position shift of the zeolite hump at 2θ range of 27–29°, with the hump’s centroid shifting from 25.4° 2θ to 29.5° in the XRD diffractogram. This observation was an indication of the greater extent of the alkali-activation process of metakaolin. Similarly, the ratio of the peak heights of Al^(5)^ and Al^(6)^ to Al^(4)^ coordination in ^27^Al MAS NMR was found to be decreasing from 0.28 to 0.006 for Na/Al ratio of 0.6 and 1.8, respectively. That is in addition to the shift in Al^(4)^ peak position from 58.2 ppm to 61.8 ppm. These results indicated the lower amounts of unreacted metakaolin that are present in high Na/Al mixes.

The position of the peak obtained from ^23^Na MAS NMR scans was found to be shifting towards more positive chemical shift values with increasing the Na/Al, indicating a higher NBO/T and a potentially lower degree of polymerization of the gel structure.

Lastly, the use of Raman spectroscopy and imaging to track the alkali-activation process of metakaolin was found to be challenging due to the highly amorphous product gel. However, our Raman data revealed that some of the peaks previously attributed to reaction products likely belong to a trace yet common Anatase impurity in the precursor metakaolin. Moreover, a unique analcime like structure was detected via Raman which warrants further investigation.

Finally, comparison of data obtained from all the techniques employed here showed a clear, non-linear relationship between extent of alkali-activation reaction and Na/Al ratio suggesting diminishing returns at higher ratios. Correlations have also been found between the results of the different techniques used. The results showed a strong correlation between the cumulative heat obtained from isothermal calorimetry and the position shift of the Si-O-T band observed by FTIR. Another strong positive correlation was found between the cumulative heat and the total weight loss of the AAM obtained by TGA. In addition, the results of XRD and NMR both showed clear comparable trend with Na/Al ratio. This was deduced by comparing the shifts of ^27^Al NMR’s Al^(4)^ coordination’s peak position and XRD’s zeolite peak position, as well as the ratios of XRD’s zeolite/anatase and ^27^Al NMR’s (Al^(5)^+Al^(6)^/Al^(4)^). These findings could guide commercial applications where alkalinity of the activator is an important parameter for performance as well as safety.

## Data Availability

The original contributions presented in the study are included in the article/supplementary material, further inquiries can be directed to the corresponding author.
